# Concurrent Waldenstrom’s Macroglobulinemia and Myelodysplastic Syndrome with a Sequent t(10;13)(p13;q22) Translocation

**DOI:** 10.3390/curroncol29070363

**Published:** 2022-06-29

**Authors:** Peter A. DeRosa, Kyle C. Roche, Victor E. Nava, Sunita Singh, Min-Ling Liu, Anita Agarwal

**Affiliations:** 1 Department of Pathology, University of Maryland Medical System, Baltimore, MD 21201, USA; 2 Department of Medicine, The George Washington University, Washington, DC 20037, USA; kyleroche@email.gwu.edu; 3 Department of Pathology, The George Washington University, Washington, DC 20037, USA; victor.nava@va.gov (V.E.N.); min-ling.liu@va.gov (M.-L.L.); 4 Department of Pathology, Veterans Health Administration Medical Center, Washington, DC 20422, USA; 5 Quest Diagnostics, Chantilly, VA 20151, USA; sunita.x.singh@questdiagnostics.com; 6 Department of Hematology and Oncology, The George Washington University, Washington, DC 20037, USA; anita.aggarwal@va.gov; 7 Department of Hematology and Oncology, Veterans Health Administration Medical Center, Washington, DC 20422, USA

**Keywords:** hematology/oncology, cytogenetics, lymphoma, myelodysplastic syndrome, pathology, genetics

## Abstract

Myelodysplastic syndromes (MDS) and Waldenstrom’s macroglobulinemia (WM) are rarely synchronous. Ineffective myelopoiesis/hematopoiesis with clonal unilineage or multilineage dysplasia and cytopenias characterize MDS. Despite a myeloid origin, MDS can sometimes lead to decreased production, abnormal apoptosis or dysmaturation of B cells, and the development of lymphoma. WM includes bone marrow involvement by lymphoplasmacytic lymphoma (LPL) secreting monoclonal immunoglobulin M (IgM) with somatic mutation (L265P) of myeloid differentiation primary response 88 gene (*MYD88*) in 80–90%, or various mutations of C-terminal domain of the C-X-C chemokine receptor type 4 (*CXCR4*) gene in 20–40% of cases. A unique, progressive case of concurrent MDS and WM with several somatic mutations (some unreported before) and a novel balanced reciprocal translocation between chromosomes 10 and 13 is presented below.

## 1. Introduction

Myelodysplastic syndromes (MDS) and Waldenstrom’s macroglobulinemia (WM) are rarely synchronous. MDS is characterized by an ineffective myelopoiesis/hematopoiesis with clonal unilineage or multilineage dysplasia and cytopenias. WM includes bone marrow involvement by lymphoplasmacytic lymphoma (LPL) secreting monoclonal immunoglobulin M (IgM) with somatic mutation (L265P) of myeloid differentiation primary response 88 gene (*MYD88*) in 80–90%. We present a case of concurrent MDS and WM, with a unique translocation and somatic mutations.

## 2. Case

**Figure 1:**
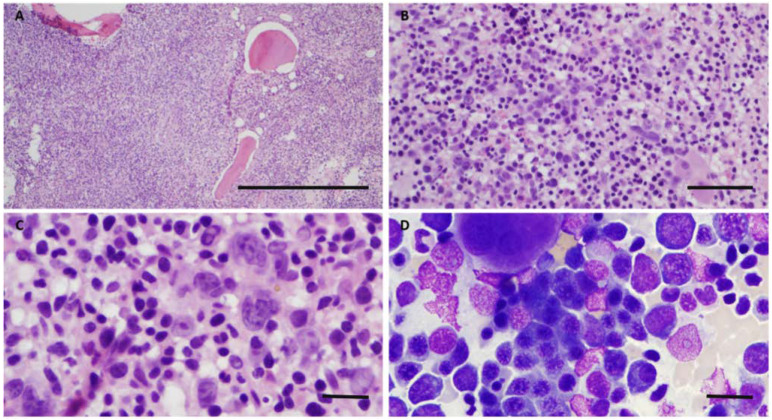
Initial diagnostic bone marrow biopsy and aspirate. The marrow is markedly hypercellular with diffuse lymphoid infiltrates consisting of predominant small lymphocytes, plasmacytic lymphocytes and rare plasma cells (H&E at 20× and 400×, respectively, in (**A**) and (**B**)). In the background, there is maturing trilineage hematopoiesis with dysmegakaryopoiesis and few blasts ((**C**). H and E at 1000×) and erythroid hyperplasia with dysplasia ((**D**) Wright-Giemsa stained aspirate at 1000×). (Scale bars = 2 mm in magnification at 20×, 50 μm at 400×, and 25 μm at magnification 1000×).

**Figure 2:**
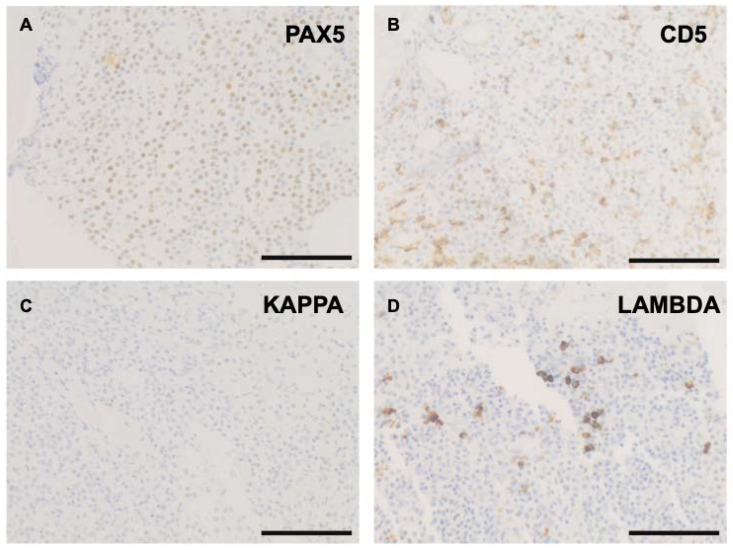
Immunohistochemistry of the initial bone marrow biopsy showing the lymphoplasmacytic lymphoma to be positive for Pax5 (**A**) and negative for CD5 (**B**) with a plasmacytic component negative for kappa (**C**) and positive for lambda (**D**). (Scale bars = 50 μm at magnification 400×).

A 71-year-old, previously healthy, African American, male, landscaper, presented in 2014 with a 2 month history of shortness of breath, intermittent light-headedness, and fatigue. Work up detected normocytic anemia (hemoglobin (Hgb) = 6.1 g/dL), thrombocytopenia, (platelets (Plt) = 111 × 10^9^/L), proteinemia (IgM = 1140 mg/dL) and monoclonal gammopathy (IgM Kappa = 0.43 g/dL). White blood cell count (WBC = 3.8 × 10^3^/µL), urine protein electrophoresis, serum free light chain studies, and bleeding/hemolytic workup were normal. A bone marrow (BM) biopsy (Figure 1) showed hypercellularity for age (95%), erythroid hyperplasia (myeloid:erythroid ratio of 1:3) with megaloblastic changes, mild left shift, megakaryocytic dysplasia with clustering and <5% blasts. A diffuse interstitial lymphoid infiltrate composed mostly of small, mature lymphocytes with plasmacytic differentiation (lymphoplasmacytes) representing approximately 30% of cellularity was noted. Diffuse moderate reticulin fibrosis (grade 1–2 on a 0–3 scale) was present (Figure 1). Flow cytometry analysis (including plasma cell and paroxysmal nocturnal hemoglobinuria panels) showed no immunophenotypic abnormalities due to aspirate sampling variation. Immunohistochemistry (IHC) revealed monoclonal, kappa-restricted lymphoplasmacytes positive for CD20, CD45, Pax5 (variably) and CD138; and negative for lambda, CD5 and CD10 (Figure 1 and Figure 2). CD34 stained rare scattered blasts. Glycophorin A and myeloperoxidase confirmed erythroid hyperplasia.

Cytogenetic analysis demonstrated a normal 46XY karyotype. PCR was negative for *JAK-2* (V617F and exons 12/13), *MPL* (W515 AND S505), *CAL-R*, *MYD88*, *CXCR-4* and *BCR*/*ABL* mutations. Fluorescent in situ hybridization analysis was negative for deletions of 5q, 7q and 20q, monosomies of chromosomes 5 and 7, trisomy of chromosome 8, and for common aberrations associated with chronic lymphocytic leukemia or multiple myeloma. Therefore, the diagnoses of low-grade B cell lymphoproliferative disorder with plasmacytic differentiation, consistent with lymphoplasmacytic lymphoma (LPL) and a concomitant myeloid neoplasm compatible with low grade MDS were made.

Lenalidomide (10 mg for 21/28 days) was started per patient’s request to avoid parenteral hypomethylating agents, and transfusion independence was achieved for 1 year (until April 2015). Afterward, pancytopenia (WBC = 2.7 × 10^3^/µL, Hgb = 7 g/dL, MCV = 75 fL, Plt = 32 × 10^9^/L) and WM (M spike = 0.53 g/dL and IgM = 1500 mg) recurred. The patient received supportive treatment with blood product transfusions and eltrombopag prior to being scheduled for a BM transplant and receiving azacytidine for pre-transplant cytoreduction. A repeat BM biopsy (August 2015) showed residual LPL with normal cytogenetics. Hemolytic workup was negative, and also demonstrated hyperproteneimia (total protein = 8.6 g/dL) with an M-spike of 1.5 g/dL and total IgM of 2.86 g/dL indicating progression of WM.

Azacytidine was stopped after 2 cycles given the development of pancytopenia secondary to WM progression in the setting of treatment with 2 doses of IVIG and 4 cycles of rituximab. The patient received maintenance rituximab therapy from 2015 to 2018 before being transitioned to ibrutinib and obinotuzumab in August, 2018, due to disease progression. This treatment regimen achieved IgM normalization prior to being discontinued in March 2019 due to persistent pancytopenia and an M spike surge.

A new BM biopsy (March 2019) revealed increased cellularity (90%) with residual low burden LPL, marked megakaryocytic hypoplasia, moderate residual reticulin fibrosis and an impressive reactive T cell infiltrate (confirmed by a negative T cell receptor rearrangement by PCR). Chromosomal analysis showed a new 20(q11.2q13.1) deletion supporting the diagnosis of residual MDS. NGS (Foundation Medicine) revealed *PTEN G132D*, B2M L15fs*41, *CD58* S212*, *CXCR4* Q318*, *FOXP1 A100*fs*50, *POT1* N514fs*7, *TNFAIP3* splice site 296-2A>G and R183* and *CXCR* Q318*. In addition, several additional variants of uncertain significance were reported: *BRCA2 D1923A* and *R2502C*, *CSF1R L125M*, *EGFR T384S*, *EP300 M2130I*, *ERBB4* V486L, *FAS D265E*, *FAM46C* L117_E122>WQEVQK, *FBXO11* V545L, *FGF23* P195S, *HIST1H1E* I80M, *KMT2A* (MLL) R2194H, *LRP1B G1691V*, *MLL2* Q3867K, *PAG1* T404S, *PDCD1* (PD-1) A263T, *PLCG2* E480K, *SMARCA1* R259G, *TBL1XR1* H441R and V210L, *TLL2* R657W and *ZNF217* A802T. All mutations were predicted as somatic based on frequency, loss of heterozygosity and copy number.

**Figure 3:**
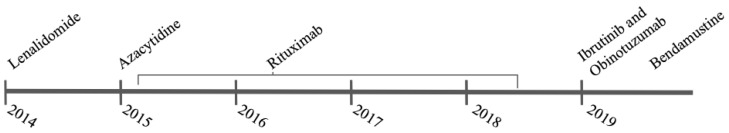
Treatment timeline during patient’s clinical course.

In August 2019, one cycle of dose-reduced bendamustine (30 mg/m^2^) and rituximab was administered, achieving improvement in blood transfusion requirements and undetectable M component. Figure 3 provides an outline of the patient’s treatment. However, severe pancytopenia (WBC = 0.5, Hgb = 8 and Pts = 20) persisted. A pre-BMT BM biopsy (November 2019) showed decreased cellularity (50%) mostly composed of reactive T cells, stable moderate reticulin fibrosis and no residual lymphoma. Corresponding cytogenetics showed a novel t(10;13)(p13;q22) translocation.

In January of 2020, the patient was hospitalized in preparation for an allogeneic BMT. However, severe pancytopenia persisted (despite 60 units of pRBCs and 20 units of Plts) and the patient developed respiratory failure requiring intubation. A transtracheal aspirate culture revealed methicillin-resistant staphylococcus aureus and he ultimately expired. The autopsy showed marked multisystemic hemosiderosis involving liver, spleen, pancreas, adrenal glands, thyroid and lymph nodes. Post mortem BM biopsy showed marked aplasia without myelofibrosis or residual LPL. The cause of death was sepsis and iron overload.

## 3. Discussion

Herein, we presented a complicated case of simultaneous MDS and WM in a 71-year-old African American male, which required multiple therapies during a 6-year prolonged course. LPL and MDS were refractory to initial lenalidomide treatment. Diverse therapeutic strategies, including ibrutinib and obinotuzumab finally achieved WM remission; however, refractory MDS persisted and a 20(q11.2q13.1) deletion was detected 4 years after treatment. The deletion of the long arm of chromosome 20, or del(20q), is a common cytogenetic abnormality in various myeloid disorders, such as primary MDS (and less frequently secondary MDS) [1,2,3], but is less common in lymphoid neoplasms, including WM [4,5,6,7,8]. Therefore, the observed del(20q) may represent primary de novo MDS and/or LPL, or a secondary therapy-related malignancy.

NGS revealed many mutations potentially representing novel therapeutic targets involving various signaling cascades: NFkB-related cell proliferation/survival (*CXCR4*, *TBL1XR1* and *TLL2*); *PI3K*/*AKT*/*mTOR*-induced protein synthesis/cell growth (*CXCR4*, *CSF1R*, *EGFR*, *ERBB4*, *FGF23*, *FOXP1*, *LRP1B*, *PAG1*, *PLCG2*, *PTEN* and *TNFAIP3*), apoptosis regulation (*B2M*,(7) *FAS*, *FBXO11*,(8) *PDCD1*/*PD-1* and *ZNF217*); maintenance of genomic stability/chromatin remodeling (*BRCA2*, *EP300*, *HIST1H1E*, *MLL*, *MLL2*, *POT1*, and *SMARCA1*); and evasion of antineoplastic immune responses (CD58). Interestingly at least fifteen novel alterations (*CXCR4* Q318*, *ERBB4* V486L, *FAS* D265E, *FAM46C* L117_E122>WQEVQK, *FBXO11* V545L, *FOXP1* A100fs*50, *LRP1B* G1691V, *MLL*/*KMT2A* R2194H, MLL2 Q3867K, *PDCD1* (PD-1) A263T, *POT1* N514fs*7, *SMARCA1* R259G, *TNFAIP3* splice site 296-2A>G, *TLL2* R657W and *ZNF217* A802T) were discovered, which may inform the complex pathogenesis of this combined malignancy or represent therapy-related secondary hits [9].

Mutations in many of these genes have been shown to be pathogenic either in WM (*B2M*, *CXCR4*, EP300, *FAM46C*, *FOXP1*, *HIST1H1E*, *LRP1B*, *MLL*, *MLL2*, *PLCG2*, *PTEN*, *TBL1XR1* and *TNFAIP3*) or MDS (*BRCA2*, *CSF1R*, *EGFR*, *EP300*, *FAS*, *FOXP1*, *MLL*, *MLL2*, *PLCG2*, *PTEN*, *POT1* and *PTEN*) [1,3,10,11,12,13,14,15], while the rest have not been reported in association with WM (*BRCA2*, *CD58*, *ERBB4*, *FAS*, *FBXO11*, *FGF23*, *PAG1*, *PDCD1*, *POT1SMARCA1*, *TLL2* and *ZNF217*) or MDS (*B2M, CD58, CXCR4, ERBB4, FAM46C, FBXO11, FGF23, HIST1H1E, PAG1, PDCD1, SMARCA1, TBL1XR1, TLL2, TNFAIP3* and *ZNF217*) yet.

Well-known druggable oncogenic targets were *PTEN, CXCR4, MLL2* and *BRCA2* [1,3,14,16]. However, further studies are necessary to fully understand the significance of these alterations and precision medicine was not attempted since the patient expired.

*PTEN* loss has been found in 3% of diffuse large B cell lymphomas but has not been reported in WM [17,18]. Although the *PTEN* G132D point mutation seen in our patient has not been functionally characterized, it has been reported in the context of PTEN hamartoma tumor syndrome [19].

*CXCR4* mutation is a frequent event in WM (30%) and is associated with survival-independent aggressive *MYD88* L256P mutated LPL [14]. However, the role of *CXCR4* in the context of wild-type *MYD88*, such as in our patient, is unknown. *CXCR4* truncation in WM has been linked with resistance to ibrutinib, but we detected *CXCR4*—Q318* after ibrutinib treatment [14].

B2M alterations, *CD58* S212*, *FOXP1* A100fs*50, *POT1* N514fs*7, *TNFAIP3* splice site 296-2A>G, R183*, *TNFRSF14* T169fs*65 have been reported in hematological malignancies and may be playing a pathogenic role in this case [7].

Approximately 5 years after diagnosis, we detected a novel balanced reciprocal translocation, t(10;13)(p13;q22) of uncertain significance. This translocation could represent novel gene fusions that may disrupt/dysregulate critical genes at the break points, or represent a chemotherapeutic induced passenger mutation. Interestingly, the closest reported translocation, t(10;13)(q21;q14) involving *CDK1* and *DGKH,* is believed to be pathogenic in acute lymphoblastic leukemia/lymphoblastic lymphoma [20,21,22,23]. The new translocation we found, was detected in unstimulated cultures, which suggests a secondary myeloid related change, or less likely, a transient post-treatment hit without significant clinical impact.

## 4. Conclusions

The development of cancer therapeutics targeting patient-specific mutational profiles remains an active area of research. We presented a unique case of synchronous WM and LPL with novel mutations in common driver genes [24] and a unique translocation, which may inform the pathogenesis or therapeutic strategies for complex cases in the future.

## Data Availability

The data presented in this study are available on request from the corresponding author.
